# Scientific discourse in the era of open science: a response to Hall et al. regarding the Carbohydrate-Insulin Model

**DOI:** 10.1038/s41366-019-0466-1

**Published:** 2019-10-04

**Authors:** David S. Ludwig, Paul R. Lakin, William W. Wong, Cara B. Ebbeling

**Affiliations:** 1 New Balance Foundation Obesity Prevention Center, Boston Children’s Hospital and Harvard Medical School, Boston, MA USA; 2 Institutional Centers for Clinical and Translational Research, Boston Children’s Hospital, Boston, MA USA; 3 USDA/Agricultural Research Service, Children’s Nutrition Research Center, Department of Pediatrics, Baylor College of Medicine, Houston, TX USA

**Keywords:** Physiology, Immunology

## Introduction

In November 2018, we presented the results of a large macronutrient feeding study at The Obesity Society’s Blackburn Symposium [1] and simultaneously in BMJ, with peer review available online [2]. In that study, total energy expenditure (TEE) measured by doubly-labeled water (DLW) at post-weight-loss and at 10 and 20 weeks of weight-loss maintenance was 200–280 kcal/day greater on a low- versus high-carbohydrate diet, consistent with the Carbohydrate-Insulin Model of obesity [3]. To facilitate scientific discourse, we made the full database immediately available on Open Science Framework.

At the Blackburn Symposium, and in subsequent comments elsewhere, Kevin Hall raised a series of concerns about our study, including the possibility of baseline instability in weight, randomization failure for other reasons, and inaccuracy of DLW due to isotope sequestration by de novo lipogenesis [4, 5]. We aimed to refute those criticisms with evidence to show baseline weight stability, a lack of difference in weight change between diet groups before and after randomization, and insignificant rates of de novo lipogenesis on relevant diets [6–8].

In their current reanalysis, Hall et al. [9] reiterate several previous concerns and raise new ones. Here, we respond to three key questions related to our study. To facilitate this response, we obtained more accurate and precise data on energy intake during the weight-loss maintenance phase of our study [10].

## What is the appropriate baseline for studying metabolism during weight-loss maintenance?

In earlier versions of this reanalysis (before Speakman joined as coauthor) [11], and elsewhere [4], Hall and Guo argue that the pre- rather than post-weight-loss measurement is the more appropriate baseline for examining diet effects on TEE. However, that approach disregards important biological variation among individuals regarding how body composition and metabolism change in response to weight loss, which averaged 9.6 kg in our study. Variation in these major confounders would introduce imprecision in statistical models involving inter-individual comparisons in this parallel design. As a general rule, baseline data should be collected as close to randomization as possible to decrease error arising from any time-varying covariate (the pre-weight-loss measurement would entail a 4-month lag until randomization). For these reasons, weight-loss-maintenance studies like Diogenes typically use the post-weight-loss timepoint.

**Fig. 1:**
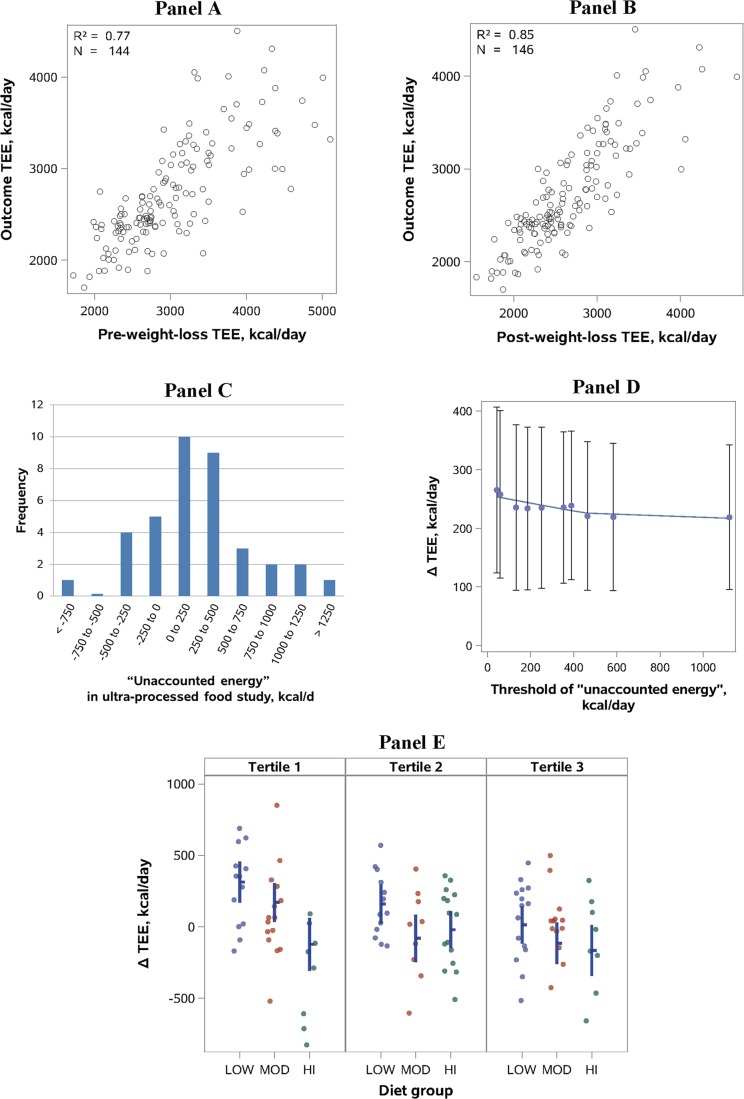
All panels involve data from our weight-loss-maintenance trial [2, 10], with the exception of **c**, which comes from Hall et al. [17]. **a, b** Correlation between TEE at baseline and outcome was stronger using the post-weight-loss versus pre-weight-loss timepoint (Meng’s *Z*-test, *p* = 0.003), demonstrating why choice of the post-weight-loss timepoint would be preferable. **c** “Unaccounted energy” in a metabolic ward study of “ultra-processed” foods [17] calculated from online data (deltabc.sas7bdat), with each participant studied on two diets for 2-week periods with DLW measurement of TEE and DXA measurement of body composition. “Unaccounted energy” (kcal/day) was calculated as energy intake (variable names: ProcAveEI, UnprocAveEI) – TEE (DLWEEProcessed, DLWEEUnproc) – change in stored energy per day (BCEBProcessed, BCEBUnproc). Only 15 of 37 observations were within ±250 kcal/day. **d** Analysis modeled after **b** in Hall et al. [9] of “unaccounted energy,” with sequential elimination of 40 (vs 81) individuals with low TEE relative to energy intake (vs low energy intake relative to TEE) in our intention-to-treat group. The observed diet effect inflates (vs deflates), demonstrating the bias introduced by postrandomization exclusion involving variables linked to the primary outcome. Because assumptions of linear regression were not satisfied (a problem that also applies to the corresponding figure in Hall et al. [9]), we used LOESS to visualize the relationship between threshold and ΔTEE. **e** Diet effect by tertiles of energy intake to TEE ratio in the Per Protocol group. Individual data indicated by circles. Mean (horizontal line) and 95% CIs (vertical bars) derived from a model adjusted for post-weight-loss TEE and other covariates (cohort, sex, age, percent weight loss, post-weight-loss weight). The difference in diet effect across the tertiles diminishes with adjustment, demonstrating the confounding present in raw data segregated by energy intake to TEE ratio. (This adjustment for baseline covariates would not remedy bias arising from postrandomization biological or measurement variation.)

We explored this issue by comparing the pre-weight-loss (Fig. 1a) or post-weight-loss (Fig. 1b) measurement of TEE with TEE measured at 10 and 20 weeks after randomization. As expected, the post-weight-loss timepoint yielded a stronger correlation. Furthermore, the results of the ANCOVA analysis by Hall et al. [9], without use of either baseline, yielded a diet effect similar to our change models with the post-weight-loss baseline.

A related concern of Hall and Guo [11] is the possibility of weight instability and ongoing metabolic adaptations during the post-weight-loss baseline measurements. However, as previously considered [6], weight change during the 15-day assessment period was small, averaging 23 g/day, with no significant difference between individuals who would be assigned to the three diet groups. Moreover, the experimental design protects against type I (false-positive) error due to ongoing metabolic adaptations (or any other prerandomization factor).

According to a basic principle of statistical analysis, the most powerful method available should be chosen to avoid type II (false-negative) error. Clearly, use of the post-weight-loss timepoint provides the most precise and least biased estimate.

## Was the change in our analysis plan involving choice of baseline proper?

Hall et al. [9] criticize us for revising the final analysis plan to the post-weight-loss baseline, after our initial registry specified the pre-weight-loss timepoint. As previously discussed [7], the pre-weight-loss specification was an inadvertent holdover from a prior crossover study [12], in which no post-weight-loss timepoint was obtained (or needed). Unlike in parallel studies, use of a pre-weight-loss baseline would not introduce major confounding in crossover studies because of the within-individual nature of the comparisons. Biological changes during weight loss for each participant, with randomization, would affect all diet periods equally.

We identified this misspecification during preparation of our final analysis plan under supervision of our statistician, made the change before breaking the data blind, and disclosed the registry change in the manuscript. An analysis with the pre-weight-loss baseline is available in online peer review [2], and we released the full database to facilitate alternative analyses.

In the CALERIE study, the final analyses were similarly “prespecified before initiation of analyses” [13], even though data had accumulated during the study [14]. Arguably the three largest macronutrient diet studies of the last decade—CALERIE, DIETFITS, and Diogenes—each made changes involving the primary outcome from initial registry posting to final publication. With the greater methodological heterogeneity of diet versus drug studies [15], registry changes and discrepancies are more the rule than the exception. Hall should appreciate this point. The registry for his only macronutrient RCT [16] did not specify a primary outcome, among other deficiencies that remain as of the 58th revision in February 2019.

Thus, our registry procedures are entirely consistent with standard practice in nutrition research and carry little risk of bias.

## Could nonadherence to the test diets in our study explain the primary finding?

Before addressing this question, the notion of “unaccounted energy” introduced by Hall et al. warrants examination. In the reanalysis, they compared reported energy intake and expenditure in our study, identifying discrepancies as violating the “physical law of energy conservation.” But this treatment disregards substantial cumulative error arising from measurement of the various components of energy balance, each with recognized imprecision and temporal variation. In the recent study by Hall et al. of ultra-processed food [17], conducted in the optimal environment of a metabolic ward, mean energy discrepancy on one diet was large (382 kcal/day) and “unaccounted energy” exceeded 250 kcal/day for most participants (Fig. 1c).

Recognizing this inherent variability and imprecision in the measurement of both energy intake and TEE, we can see why exclusions involving their difference or ratio (as in Fig. 1b of Hall et al.) would produce highly misleading results. Whereas individuals with low energy intake relative to TEE might have been nonadherent (i.e., unobserved food intake), they would also tend to be at the upper end of the natural distribution for TEE (related to true biological differences or randomly distributed measurement variation). Therefore, eliminating them would deplete the cohort of those with the greatest TEE denominator, deflating the diet effect.

We can demonstrate this phenomenon in three ways. First, we conducted the converse analysis, sequentially eliminating individuals with “unaccounted energy” arising from low TEE to energy intake (here, TEE resides in the numerator). As illustrated in Fig. 1d, the diet effect now increases with the progressive threshold because individuals at the lower end of the TEE distribution are eliminated, leaving a residual cohort enriched for hyper-responders. However, these models, involving postrandomization variables inextricably linked to the outcome, violate a basic principle of statistical inference and should be discarded as fatally flawed.

Second, we divided the per protocol (weight stable) group into tertiles, based on the ratio of energy intake to TEE (Fig. 1e). In an unadjusted model, those in the lowest tertile (i.e., those eliminated in the analysis of Hall et al.) demonstrated a substantially larger diet effect. However, they were also more likely than those in the other two tertiles to have a baseline TEE above the median (OR 2.7 [95% CI 1.2–6.1], *p* = 0.02). With adjustment for baseline TEE and other relevant covariates, the differences between the tertiles for diet effect diminished.

Hall et al. modeled CO_2_ production (rCO_2_) in our cohort to circumvent the need for respiratory quotient (RQ), deviating from well-established DLW methodology and introducing severe bias against the low-carbohydrate diet. Because food quotient (FQ) equals RQ during weight (and body composition) stability, as applies to our per protocol group, a third approach is to conduct a sensitivity analysis examining how varying degrees of nonadherence would affect FQ and thereby TEE. As shown in Table 1, the low- versus high-carbohydrate diet comparison remained statistically significant through 50% nonadherence. Of particular interest, the diet effect relative to carbohydrate proportion remained remarkably stable throughout the range of assumed nonadherence, and consistently above the hypothesized 50 kcal/day for every 10% decrease in the proportion of energy as carbohydrate [3]. Moreover, among participants in the lowest tertile of energy intake to TEE (for whom estimates of FQ may be least accurate), the unadjusted change in rCO_2_ was itself significantly greater on the low- versus high-carbohydrate diet (10.3 vs −47.0 L/day, *p* = 0.01). That is, the diet effect on TEE in this subgroup was so large as to require no assumptions about FQ, providing further evidence against nonadherence as an explanation for study findings.

**Table 1:** Sensitivity analysis of TEE. This analysis examines how potential nonadherence could influence the diet effect on TEE in the per protocol (weight stable) group, considering how FQ (used in DLW methodology) changes with macronutrient ratio

	Degree of nonadherence (proportion of total)^a^						
	0%	10%	20%	30%	40%	50%	60%
FQ low-carbohydrate diet	0.7880	0.7938	0.7996	0.8054	0.8112	0.8170	0.8228
FQ high-carbohydrate diet	0.9040	0.8982	0.8924	0.8866	0.8808	0.8750	0.8692
TEE diet effect^b^ kcal/day	280^c^	249^c^	219^c^	188^c^	158^c^	127^c^	97
TEE diet effect kcal/day per 10% decrease in carbohydrate	70	69	68	67	66	64	60

^a^Assumes that any foods eaten off protocol for both low-carbohydrate (20% carbohydrate, 60% fat) and high-carbohydrate (60% carbohydrate, 20% fat) diet groups reflected the macronutrient composition of the moderate-carbohydrate (40% carbohydrate, 40% fat) diet group

^b^Difference between low- and high-carbohydrate diet groups, minimally adjusted for cohort, as per analyses in BMJ [2]

^c^Remained statistically significant at *p* ≤ 0.05

As stated in the BMJ article, our preliminary estimates of energy intake, used by Hall et al., “would tend to selectively underestimate those with high energy expenditure” and were not intended to be definitive. With more precise and accurate data [10], we found that energy requirements for weight stability (i.e., by calorie titration) showed a similar magnitude of effect (≈200–300 kcal/day) and hierarchical order (low > moderate > high carbohydrate) among diets as TEE, as predicted by the Carbohydrate-Insulin Model. Due to imprecision involved in these (and all) methods for determining outpatient energy intake and expenditure, the magnitude of effect should be interpreted cautiously.

## Other issues

Hall et al. set a high bar for the Carbohydrate-Insulin Model by stating that “[p]roponents of low-carbohydrate diets have claimed that such diets result in a substantial increase in … [TEE] amounting to 400–600 kcal/day”. However, the original source for this assertion, Fein and Feinman [18], characterized this estimate as a “hypothesis that would need to be tested” based on extreme assumptions about gluconeogenesis, with the additional qualification that “we [do not] know the magnitude of the effect.” An estimate derived from experimental data—and one that would still hold major implications for obesity treatment if true—is in the range of 200 kcal/day [3]. At the same time, they set a low bar for themselves, citing a 6-day trial [16] (confounded by transient adaptive responses to macronutrient change [3]) and a nonrandomized pilot study [5] (confounded by weight loss [8]) as a basis for questioning DLW methodology. Elsewhere, Hall interpreted these studies as sufficient to “falsify” the Carbohydrate-Insulin Model [19]—but they do nothing of the kind. Indeed, a recent reanalysis of that pilot study suggests an effect similar to ours (≈250 kcal/day) [20].

Finally, we agree with Hall et al. that the component(s) of energy expenditure that might underlie our findings remain unknown. The diet effects on resting energy expenditure (REE) and moderate- to vigorous-intensity physical activity were of borderline significance in the hypothesized direction (*p* = 0.06–0.09) for pair-wise comparisons. Because individual components of energy expenditure might each contribute <100 kcal/day to the diet effect, our study lacked power to examine these secondary outcomes. In our crossover study [12], we found a significantly greater REE on a very-low-carbohydrate versus low-fat (high-carbohydrate) diet in the fasting state, when the thermic effect of food would have dissipated.

## Conclusion

We aim to show that the latest series of criticisms by Hall et al., like those previously addressed, have little merit. Contrary to their claims, the data from our BMJ study, together with new data on energy intake, provide substantial support for the Carbohydrate-Insulin Model. Nevertheless, we recognize that these results require replication and that the relative advantages of dietary carbohydrate- versus fat-restriction on a population basis have not been established.

Of course, debate and criticism lie at the heart of science and should be encouraged, including with public posting of full databases. However, in the new era of open science, with widespread availability of raw data, the inevitable deficiencies in trials will be on full display. To minimize distraction and promote constructive discourse, it will be critical to distinguish inconsequential discrepancies and omissions from flaws that pose high risk of bias. Thus, we must all resist the admittedly natural tendency to hold a double standard for studies that support versus oppose our own views.

## Data Availability

The protocol and data set for the original trial findings study are available at Open Science Framework (https://osf.io/rvbuy/). New primary data on energy intake and body composition will be posted upon publication of the related findings in a peer-review journal.
